# Determinants of Timely Presentation for Birth Dose Vaccination at an Immunization Centre in North-central Nigeria

**DOI:** 10.5334/aogh.725

**Published:** 2019-03-01

**Authors:** Rasheedat Ibraheem, Mohammed Abdulkadir, Moshood Akintola, Muhammed Adeboye

**Affiliations:** 1 University of Ilorin, NG; 2 University of Ilorin Teaching Hospital, Ilorin, Kwara, NG; 3 General Hospital, Ilorin, NG

## Abstract

**Background:**

Timely receipt of immunization is an essential prerequisite to ensure early protection of the child. However, a low proportion of children in Nigeria benefit from the timely administration of the birth dose vaccines.

**Objectives:**

These were identification of factors associated with timely presentation and reasons for presentation beyond 24 hours at an immunization centre in Ilorin, Nigeria.

**Method:**

A descriptive cross-sectional study involving 480 mother-infant pairs was conducted at an immunization centre. Socio-demographic, antenatal care (ANC) and delivery details, infant’s birthday and day of presentation for vaccination were recorded. Logistic regression was used to identify factors associated with time to presentation within day one.

**Findings:**

239 (49.8%), 421 (87.7%) and 454 (94.6%) babies were vaccinated within days one, seven and 14 respectively. Post-secondary education level of mothers (OR = 3.60; 95% C.I: 1.30–9.91), antenatal care attendance (OR = 9.55; 95% C.I: 1.75–52.12), and hospital delivery (OR = 6.36; 95% C.I: 1.33–30.38) were associated with presentation within day one. Having correct knowledge of the immunization schedule increased the odds of early presentation by three times, p = 0.025. The commonest reason for presentation after day one for vaccination was weekend/public holiday delivery identified in 83 (35.2%) mother-infant pairs.

**Conclusion:**

Hospital delivery, attendance at antenatal care, postsecondary education and knowledge of the immunization schedule were factors associated with timely presentation for birth dose vaccination. Strategies to improve timeliness of the birth dose vaccination should target babies delivered outside the hospital as well as during weekends in the hospital. Also, inclusion of immunization into the health education curriculum of schools could be beneficial.

## Introduction

Despite the proven effectiveness of immunization as a tool for reducing some infectious disease-related morbidity and associated mortality, it is an intervention that is still underutilized in developing countries. In 2016, a nationwide study involving adults and children identified the prevalence of Hepatitis B among children aged less than 10 years as 9.8% and an overall prevalence of 12.2% [1]. This prevalence rate is greater than 8% and identifies Nigeria as a highly endemic area for the disease [2]. The World Health Organization (WHO) reported the median (inter-quartile range) incidence of tuberculosis amongst children in Nigeria aged less than fourteen years as 56,000 (34,000–77,000) cases [3], while the mortality rate attributable to tuberculosis for under-five children was estimated at 40–80 deaths/100,000 population [4]. Poliomyelitis causes irreversible paralysis, and while significant achievements have been made in Nigeria toward its elimination, four cases of wild poliovirus were reported in 2016 after a two-year disease-free period [5]. Considering the burden of these vaccinable, preventable diseases and their public health importance, early administration of these vaccines to the infection-naïve neonates becomes critical. Protection against vaccinable, preventable diseases can be maximized when the vaccine antigens are received as and when due because timely receipt of immunization is an essential prerequisite to ensure early protection of the child [6].

In Nigeria, infants receive a single dose of Bacillus Calmette-Guerin (BCG), hepatitis B vaccine (HepB-BD) and oral polio vaccine (OPV^0^) at birth [7,8]. The national guidelines recommend these vaccines are preferably given to an infant within 24 hours, and up to 14 days post-delivery, although BCG could still be given up till 12 months of age [8]. The birth dose of the hepatitis B vaccine introduced in Nigeria stems from the recommendation for countries with high burden of Hepatitis B to receive an early dose of the vaccine. A meta-analysis had indentified that babies who receive the HepB-BD compared with those who didn’t were 3.5 times less likely to be infected when born to hepatitis b virus (HBV) positive mothers [9]. Furthermore, 80–90% of HBV infection acquired at birth would progress to chronic disease compared to less than 5% of infection acquired in adulthood [10,11], thus emphasizing the importance of the HepB-BD in a highly endemic country to reduce perinatal spread.

Timeliness of birth dose vaccination remains a major problem in developing countries with weak immunization systems [12,13,14,15,16,17]. A recent Nigeria immunization coverage survey has shown a decline in the national BCG immunization coverage from 76% in 2010 to 53% in 2016, with regional differences as the Northwest region had the lowest coverage of 30.0% while the Southeast zone had 90.1% [18]. The 2016–2017 multiple indicator cluster survey identified that the percentage of children aged 12–23 months who had been vaccinated with BCG, HepB-BD at birth and OPV^0^ in Kwara State, Nigeria, were 72.1%, 46.9% and 61.2%, respectively, whereas those that received these vaccines at the recommended time were 13.3%, 4.3% and 4.8%, respectively [18]. Miyahara et al., in the Gambia [17], identified the proportion of children commenced on the birth dose vaccination within 24 hours and seven days to be 1.1% and 5.4%, respectively. A 2009 study by Sadoh et al. in Benin, Nigeria, reported less than 50% of infants had received BCG within two weeks of delivery [19]. In another Nigerian study by Sadoh et al., they reported a timeliness of 1.3% and 43.1% within 24 hours and seven days, respectively, for the birth dose vaccination [16]. Some reasons adduced for the delays in receiving birth dose vaccination include cost considerations, poor maternal education, unavailability of vaccines and a lack of awareness of benefits of the vaccines [19,20].

Considering the low rates of timeliness and poor immunization coverage in Nigeria, it is important to identify key determinants of early presentation for the birth dose vaccinations. Thus, the current study aimed to identify the timeliness of presentation for birth dose vaccination, factors that influenced timely presentation as well as the reasons for presentation beyond 24 hours at an immunization centre in Ilorin, Nigeria.

## Method

### Study design and setting

A descriptive cross-sectional study was conducted at the immunization centre of General Hospital, Ilorin. The hospital, located in Ilorin West Local Government Area (LGA) of Kwara State, provides health care at the primary and secondary level and serves as a referral centre within the state. Ilorin, the capital city of Kwara State, situated in the North Central geopolitical zone of Nigeria, has a population of 1,049,168 based on the 2006 census with a projected annual growth rate of 2.3%, whereas Ilorin West LGA has a population of 519,927 with the population of children aged less than five years of 103,985 [21]. Kwara State has a neonatal mortality rate of 27 per 1000 live births and an infant mortality rate of 40 per 1000 live births [18]. The percentage of women that attend antenatal care in the State was identified to be 74.8%, whereas the percentages of deliveries attended to by a skilled birth attendance and those with health facility delivery were 63.8% and 61.6%, respectively [18].

The immunization centre provides vaccination to infants from Monday to Friday, except during public holidays. The State Primary Health Care Development Agency (PHCDA) supplies the vaccines to each local government area in the state, and the vaccines are collected from the central area twice to thrice a week for utilization at the immunization centre. Multi-dose vials for the OPV (10 doses/vial), hepatitis B (10 doses/vial) and BCG vaccines (20 doses/vial) are provided and administered to the infant at no cost to the parent. Services rendered at the immunization unit of the hospital include vaccination, growth monitoring, nutrition education and general health education.

### Sample size determination

The formula used for estimating the minimum sample size required for the study was “n (z^2^pq)/d^2^” where: n = the desired sample size; z = the standard normal deviate set at 1.96, which corresponds to 95% confidence interval; p = the proportion in the target population estimated to present for vaccination within seven days (estimated to be 43.1% from an earlier study in Nigeria [16]); q = 1.0–p; and d = degree of accuracy desired, which is 0.05. The minimum sample size calculated was 392, however 480 mother-baby pairs were recruited.

The eligibility criteria consisted of mothers/caregivers bringing their newborns for the birth dose vaccine and consent to participate in the study.

### Data collection instrument

This was a semistructured interview based questionnaire which was deployed in either English or Yoruba language by two research assistants, with an average time to completion of five minutes. Every mother-baby pair who presented at the immunization centre and who satisfied the eligibility criteria was subsequently enrolled till the sample size was reached, and this lasted for a period of five months (August–December 30, 2016). The socio-demographic details of each mother-infant pair presenting for vaccination, such as gender of child, age of mother, religion, marital status, level of education and occupation of the child’s parents, were recorded in the study proforma. The social class of the infant was derived using the method described by Oyedeji [22] Responses on whether mother had antenatal care (ANC), as well as place of delivery of the baby, were sought and recorded. Responses on the birth order of the infant, the number of mother’s children, as well as a history of previous and complete vaccination appointments for elder siblings were documented. Responses on the mother’s knowledge of the timing and number of visits for routine immunization for an infant were also recorded. The infant’s date of birth and the date the baby was brought to the immunization centre for the birth dose vaccination were recorded. For infants whose vaccination date was later than 24 hours after the date of birth, responses were sought from the mother/caregiver on the reason for the delay in presentation.

After obtaining these details, mothers were educated about the importance of immunization, as well as the number and timing of each immunization appointment.

### Data analysis

Data was analyzed using the IBM SPSS version 20.0 (IBM Corporation, Virginia, USA, 2011). The difference between the date of birth and the day of presentation was calculated. The interval (in days) to presentation was derived by calculating the difference between the date of birth of the infant and the day of presentation for the birth dose vaccinations. The day of birth was recorded as day 0 and the day after delivery as day 1. Time to presentation was identified for three periods namely within 24 hours (day 0 and day 1), within seven days (day 0 till day seven) and within 14 days (day 0 till day14). The national guidelines recommend receipt of birth doses preferably within 24 hours, and up to 14 days, however previous studies on timing of birth vaccinations in the country had utilized the time frames of 24 hours [12,16], seven days [12,16] and 14 days [19], hence the rationale for the choice of time intervals. Also, the time to presentation was divided into four mutually exclusive groups as follows: within day 1, between day two and day seven, days eight to 14 and greater than 14 days.

Continuous variables were expressed as mean and standard deviation (SD) when normally distributed and as median (inter-quartile range) if not normally distributed, while categorical variables were expressed as frequencies and percentage. The chi-square (χ*^2^*) test and student t-test were used to identify significant differences among categorical variables and continuous variables, respectively. Variables with a *p*-value less than 0.10 on univariate analysis in Table 4 were included in the multinomial logistic regression model to identify factors associated with presentation of infants by their mothers for the birth dose vaccination for the different time periods. A *p*-value of less than 0.05 was considered statistically significant.

### Ethical review

The study protocol was reviewed and approved by the ethical board of the Kwara State Ministry of Health. Informed consent was obtained from the caregiver after clear explanation about the study had been given to the individual. No incentives were provided for participating in the study.

## Results

A total of 503 babies were brought for first-time vaccinations; 480 (95.4%) mother-infant pairs met the eligibility criteria and were enrolled, and 23 (4.6%) mother-infant pairs were not enrolled because those that brought the babies were unable to provide the needed information or consent.

The mean (SD) age of the 480 mothers enrolled was 28.4 (4.7) years, ranging between 17–45 years. The male infants were 245 (51.0%), and the females were 235 (49.0%). Six (1.3%) babies were from a single household with unmarried mothers, 470 (97.9%) babies had parents who were married and four (0.8%) children were from a divorced home. Islam was the religion of 346 (72.1%) mothers, and 134 (27.9%) mothers practiced Christianity. The other socio-demographic details and delivery characteristics of the mother-infant pairs are shown in Table 1.

**Table 1:** Socio-demographic, antenatal and delivery characteristics of the mother-infant pairs.

Variable	Frequency N = 480	Percentage

**Maternal educational level**		
None/primary	8	1.7
Secondary	164	34.2
Post-secondary	308	64.2
**Maternal age group (years)**		
<21	21	4.4
21–25	124	25.8
26–30	194	40.4
31–35	108	22.5
>35	33	6.9
**Antenatal care attendance for index pregnancy**		
Yes	449	93.5
No	31	6.5
**Place of delivery**		
Home	23	4.8
Traditional birth attendant (TBA) home	4	0.8
Private hospital	51	10.6
Government hospital	384	80.0
Church	18	3.8
**Social class of infant**		
1	17	3.5
II	254	52.9
III	206	42.9
IV	3	0.6
**Birth order**		
First	196	40.8
Second–Third	220	45.8
Fourth–Fifth	58	12.1
Sixth–Seventh	6	1.3

The median (inter-quartile range) interval between birth and receipt of the birth dose vaccination was 2.0 (1.0–4.0) days. The maximum interval was day 127, and the minimum was day 0. Two hundred thirty-nine (49.8%) babies received their vaccine within day one after delivery, 182 (37.9%) babies between the second and seventh day and 33 (6.9%) infants between the eighth and 14th day, whereas 26 (5.4%) babies were presented later than 14 days for receipt of the first dose of vaccines.

Of the 241 babies brought beyond day one of life for immunization, 236 (97.9%) mothers stated the reason for the presentation beyond day one. Table 2 shows the most common reasons for presenting after day one of delivery were delivery during a weekend/public holiday and Friday evening delivery/discharge, identified for 83 (35.2%) and 26 (13.6%) infants, respectively.

**Table 2:** Reasons stated for presentation after day one for birth dose vaccination.

Reason for presentation beyond Day 1	Frequency N = 236	Percent	Interval between delivery and presentation for vaccination (Days)	

			Median	Range

***Access to vaccine related***				

Weekend/public holiday delivery	83	35.2	3.0	2–26
Friday evening delivery/discharge	32	13.6	3.0	2–18
Private hospital delivery	20	8.5	3.5	2–28
Fixed days for immunization at place of delivery	10	4.2	2.5	2–11
Home delivery	7	3.0	12.0	5–33
Delivery at Church	7	3.0	8.0	2–44
Delivery at TBA home	3	1.3	3.0	2–8
No nearby place for vaccination	3	1.3	17.0	14–42
Vaccine unavailable at initial place of presentation	1	0.4	5.0	5
***Mother related***				

Ill mother	18	7.6	6.5	2–57
Had cesarean section	14	5.9	3.5	2–17
Could not afford transport cost	5	2.1	11.0	7–50
Unaware that centre provided vaccination every weekday	3	1.3	7.0	2–9
Needed rest after delivery	3	1.3	2.0	2–8
Deceased mother	1	0.4	2.0	2
Mother in school	1	0.4	6.0	6
***Infant related***				

Ill baby	23	9.7	4.0	2–19
Prematurity	2	0.8	88	49–127

(TBA: traditional birth attendant home).

Of the 449 mothers who attended ANC, 299 (66.6%) had post-secondary level of education, and 150 (33.4%) mothers had secondary school or lower educational level, p =< 0.01 Of the 244 mothers who correctly identified the NPI schedule, 155 (63.5%) mothers had post-secondary education, whereas 89 (36.5%) mothers with secondary school or lower educational level identified it correctly, p = 0.77.

Comparing those who presented within day one and those who presented after day one, hospital delivery was the sole determinant of presentation because infants delivered in the hospital had an almost five times increased odds of being vaccinated compared with infants not delivered in the hospital (Table 3).

**Table 3:** Factors associated with timely presentation (within day 1) for birth dose vaccination.

Parameter	Time to presentation		Crude OR (95% C.I.)	p*	Adjusted OR (95% C.I.)	p**

	≤ day 1 N = 239 n (%)	> day 1 N = 241 n (%)				

**Gender**						
Male	124 (50.6)	121 (49.4)	1.07 (0.75–1.53)	0.714		
Female	115 (48.9)	120 (51.1)				
**Religion**						
Islam	179 (51.7)	167 (48.3)	1.32 (0.89–1.97)	0.171		
Christianity	60 (44.8)	74 (54.2)				
**Mother’s age (years)**						
Mean (SD)	28.26 (4.76)	28.48 (4.66)	–	0.606δ		
**Mother’s level of education**						
Postsecondary	168 (54.5)	140 (45.5)	1.71 (1.17–2.49)	0.005	1.44 (0.99–2.13)	0.072
≤Secondary	71 (41.3)	101 (58.7)			1(reference)	
**Social class of child**						
Upper (I, II)	144 (53.1)	127 (46.9)	1.36 (0.95–1.99)	0.095		
Lower (III, IV)	95 (45.5)	114 (54.5)				
**Birth order of infant**						
First	100 (51.0)	96 (49.0)	1.09 (0.96–1.56)	0.655		
≥Second	139 (56.7)	145 (43.3)				
**ANC**						
Yes	235 (52.3)	214 (47.7)	7.41 (2.55–21.53)	<0.001	2.50 (0.73–8.52)	0.143
No	4 (12.9)	27 (87.1)			1(reference)	
**Hospital delivery**						
Yes	233 (57.5)	202 (42.5)	7.50 (3.11–8.07)	<0.001	4.64 (1.73–12.45)	0.002
No	6 (13.3)	39 (86.7)			1(reference)	
**Correctly states NPI schedule**						
Yes	116 (47.5)	128 (52.5)	0.83 (0.58–1.19)	0.316		
No	123 (52.1)	113 (47.9)				

δ = p-value derived from independent sample t-test; * = chi-square derived p-value; ** = p-value derived from logistic regression analysis; OR (95% C.I.) = odds ratio 95% confidence interval.

Table 5 shows that mothers who could correctly state the NPI schedule were three times more likely to bring their babies for vaccination during the three intervals compared to mothers with an inaccurate knowledge of the NPI schedule. Maternal post-secondary education, antenatal care attendance, and hospital delivery increased the odds of presentation within one day post delivery by four times, six times and ten times respectively, each p < 0.05. For presentations between the 2^nd^ and 7^th^ day, mothers with postsecondary educational level, and who attended antenatal care had a six times and three times increased odds respectively of bringing their infants for immunization during this interval, each p < 0.05.

**Table 4:** Univariate analysis showing factors affecting time to presentation at different intervals for receipt of birth dose vaccines.

Parameter	Time to presentation for birth dose vaccines (days)				p*

	≤ 1 N = 239 n (%)	2–7 N = 182 n (%)	8–14 N = 33 n (%)	> 14 N = 26 n (%)	

**Gender**					
Male	124 (50.6)	94 (38.4)	14 (5.7)	13 (5.3)	0.780
Female	115 (48.9)	88 (37.5)	19 (8.1)	13 (5.5)	
**Religion**					
Islam	179 (51.7)	122 (35.3)	27 (7.8)	18 (5.2)	0.179
Christianity	60 (44.8)	60 (44.8)	6 (4.4)	8 (6.0)	
**Mother’s age (years)**					
Mean (SD)	28.26 (4.76)^ab^	29.02 (4.65)^b^	26.73 (4.69)^a^	26.92 (3.97)^a^	0.018**
**Mother’s level of education**					
Post-secondary	168 (54.5)	120 (39.0)	12 (3.9)	8 (2.6)	<0.001
≤Secondary	71 (41.3)	62 (36.0)	21 (12.2)	18 (10.5)	
**Social class of child**					
Upper (I, II)	144 (53.1)	104 (38.4)	11 (4.1)	12 (4.4)	0.021
Lower (III, IV)	95 (45.5)	78 (37.3)	22 (10.5)	14 (6.7)	
**Birth order of infant**					
First	100 (51.0)	69 (35.2)	12 (6.1)	15 (7.7)	0.253
≥Second	139 (48.9)	113 (39.8)	21 (7.4)	11 (3.9)	
**ANC**					
Yes	235 (52.3)	170 (37.9)	27 (6.0)	17 (3.8)	<0.001
No	4 (12.9)	12 (38.7)	6 (19.4)	9 (29.0)	
**Hospital delivery**					
Yes	233 (53.6)	161 (37.0)	24 (5.5)	17 (3.9)	<0.001
No	6 (13.3)	21 (46.7)	9 (20.0)	9 (20.0)	
**Correctly states NPI schedule**					
Yes	116 (47.5)	101 (41.4)	19 (7.8)	8 (3.3)	0.076
No	123 (52.1)	81 (34.4)	14 (5.9)	18 (7.6)	

* = chi-square derived p-value; ** = *F*-value derived from ANOVA. ^a, b^ Duncan multiple range test shows that means with the same letter are not statistically different at p < 0.05.

**Table 5:** Multivariate logistic regression of factors affecting time to presentation for receipt of birth dose vaccines (earlier intervals) compared with those presenting after day 14.

Parameter	Time to presentation for birth dose vaccines					

	≤1 day		2–7 days		8–14 days	

	OR (95% C.I.)	*p*	OR (95% C.I.)	*p*	OR (95% C.I.)	*p*

**Mother’s age (years)**	1.05 (0.95–1.17)	0.328	1.09 (0.98–1.21)	0.102	0.99 (0.88–1.12)	0.880
**Correctly states NPI schedule**						
Yes versus No	3.08 (1.16–8.23)	0.025	3.68 (1.38–9.83)	0.009	3.77 (1.20–11.84)	0.023
**ANC attendance**						
Yes versus No	9.55 (1.75–52.12)	0.009	5.78 (1.27–26.28)	0.023	3.99 (0.69–22.82)	0.125
**Hospital delivery**						
Yes versus No	6.36 (1.33–30.38)	0.022	1.67 (0.39–6.97)	0.494	0.74 (0.15–3.74)	0.709
**Social class of child**						
Upper versus Lower	0.82 (0.31–2.16)	0.700	0.70 (0.26–1.84)	0.475	0.44 (0.13–1.43)	0.173
**Mother’s level of education**						
Postsecondary versus ≤Secondary	3.60 (1.30–9.91)	0.013	3.29 (1.18–9.13)	0.022	1.45 (0.42–4.97)	0.555

OR (95% C.I.) = odds ratio 95% confidence interval.

## Discussion

The proportion of children presenting within 24 hours and seven days for immunization of 49.8% and 87.8% in the current study is higher than the 1.3% and 43.1% recorded in Benin Nigeria [16], and 1.1% and 5.4% in the Gambia [17], respectively. Our findings are much higher than the reported birth dose coverage at the recommended time for BCG, HepB-BD and OPV^0^ of 13.3%, 4.3% and 4.8%, respectively, among children in Kwara State [18], and thus may not be truly representative of the findings in the state. The higher figure recorded may be partly explained by the fact that the immunization centre in the current study gives vaccination all weekdays as against the practice of giving BCG only on Fridays in the Benin study which accounted for one-third of the reason given for delay in presenting for immunization in the Benin study as mothers waited for the day BCG was scheduled. Also, vaccinations were done once or twice a week in the Gambia study at a specific reproductive and child clinic (RCH) or outreach clinic, which could also account for the lower proportion identified because mothers would have to wait for the specific vaccination day. The implication of these findings is that everyday vaccinations at immunization centers would increase the timeliness of receipt of birth dose vaccinations. Although the national guideline recommends up till 14 days for administration of HepB-BD [18], it is known that the effectiveness of the HepB-BD in prevention of perinatal transmission of HBV decreases with delay in administration of the vaccine [2]. Therefore the low proportion of children presenting within 24 hours of 49.8% and 44.7% between the second and 14th day poses a significant concern in a highly endemic region because it means only half of the infants were likely to have the maximal protection.

Timing of delivery and discharge were major contributors to delays in receipt of the birth dose vaccines as the majority of the infants (almost 50%) presented beyond day one for vaccination in the current study had been delivered during a weekend, Friday evening, or public holiday such that the parents needed to wait for a working day during the week to bring the infant for the required vaccination. The health workers at government-owned vaccination centers do not work on weekends and during public holidays, such that babies delivered or discharged during these periods presenting for vaccination will not have access to it. Following this delay due to the weekend or public holiday, mothers may further delay the presentation of the infant for vaccination due to forgetfulness, need to rest and lack of perception of the importance of timely receipt of vaccines. Therefore, vaccination of babies during all weekdays as well as weekends would improve the timeliness of receipt of birth dose vaccines and ensure reduced missed opportunities for vaccination. Another major reason for vaccination of babies after 24 hours in the current study was ill health of the mother, which is similar to the report by Sadoh et al. [16]. The fact that there is a delay in immunization of infants of ill mothers is a missed opportunity.

In order to improve the timeliness of birth dose vaccination, there is a need to educate health caregivers of ill mothers postpartum to enquire and ensure the infants of such mothers are vaccinated. Also, another potential avenue for increasing timeliness of vaccination could be via immunization in delivery suites after resuscitation of newborns. Training of the health care providers who deliver the infants on vaccination of babies immediately after delivery is an intervention toward improving timeliness of birth dose vaccines, which has been successfully implemented [23]. Non-availability of the vaccines at the place of delivery was also a major contributor (15%) to delays in presentations for vaccination beyond day one in the current study which brings to fore the role of the health caregiver/delivery attendants in ensuring that the infants are taken for vaccination. There is the tendency for the caregiver to either delay or forget about the vaccination of the infant if the importance of vaccination hasn’t been emphasized.

The fact that hospital delivery was associated with timely presentation for vaccination within a day in the current study is consistent with the report from Benin [16] and the Republic of Kiribati [24]. Institutional delivery rates and skilled birth attendance rates have been identified to correlate positively with the HepB-BD in the WHO African region, which further supports our findings of hospital delivery as a key factor for early receipt of vaccination [25]. The current finding however differ from the report from the Gambia [17], where hospital delivery was not associated with timely presentation for vaccination. The contrasting findings in the Gambian study compared with the others may be explained by the fact that only designated reproductive and child health clinics or outreach clinics could give immunizations either once or twice a week irrespective of the hospital where the baby was delivered, thus eliminating any advantage of ease of access to vaccination if the hospital was vaccinating babies delivered there, as is seen in the present study.

The implication of the current finding is that strategies to improve timeliness of birth dose vaccination should be targeted at the faith home (church) and traditional birth attendants (TBA) deliveries to ensure babies delivered at places with lack of access to vaccinations are promptly taken for immunization. Indeed, it has been identified that THE majority of the deliveries, especially in the northern part of Nigeria, are assisted by the traditional birth attendants or relatives at home [18]. A focused education of the community health extension workers (CHEW) who participate in the community-based newborn care to inculcate the practice of emphasizing immunization of babies to the mothers would also help improve timeliness of the birth dose vaccine. A recent study incorporating linkages between village health volunteers, the health workers and the pregnant women improved the timely birth dosage vaccination [24]. Such a model could be adopted toward improving the presentation time of babies delivered at home.

Antenatal care attendance was a significant determinant of mothers who presented between day one and day seven. Health talk is one of the services provided during antenatal care, and studies have shown this platform to be a source of information on immunization to mothers [24,26,27,28,29]. Despite delivery outside the hospital, attendance at antenatal care would equip the mother with the information on immunization, and she would therefore be likely to promptly take her baby for vaccination. Improvement in maternal access to antenatal care may therefore improve the timeliness of the birth dose vaccination.

Educated mothers have been shown to have good health-seeking behavior for their children [30], which is supported by the findings in the current study of post-secondary educational level of mothers as a significant determinant of timely presentation for birth dose vaccination. This finding had been reported from other studies [16,17,31,32]. The role of health education is further buttressed by the finding in this study that mothers who identified the expected timing and number of vaccination appointments were three times more likely to present early compared to those who gave wrong responses. Educating parents about the importance of timely birth dose vaccinations has been found to improve the timeliness of vaccination [23]. These findings could guide advocacy efforts on improving timeliness of infant immunization to focus on the less educated. The importance of immunization as a public health intervention could be introduced into the health education curriculum for secondary schools. Ensuring timely receipt of the birth dose vaccines is germane to a reduction in the pool of susceptible children to diseases such as tuberculosis and hepatitis and is therefore a step toward reducing the burden associated with these disease conditions.

A major strength of the current study is the fact that it was done in a centre where vaccinations are done every weekday with readily available vaccines, and thus we were able to identify that the unavailability of vaccinations during weekends and public holidays is a major contributor to delays in vaccinations. To avoid such missed opportunities, measures should be put in place to ensure the vaccination of children delivered at such periods.

### Conclusion

Hospital delivery, attendance at antenatal care, post-secondary education and knowledge of the immunization schedule were factors associated with timely presentation for birth dose vaccination.

### Limitation

A major identified limitation of the current study is the fact that it was undertaken in a single center where deliveries are taken, with babies being brought for immunization from the wards which precludes its applicability to the subregion. Thus, there is a need for further study at centers where deliveries are not taken, preferably a multicentered study in the different geopolitical zones of the country. Another limitation is the population enrolled is not representative of Kwara State because the social class and vaccination coverage of the enrollees are higher than the corresponding characteristics of the state.

### Recommendations

Strategies to improve timeliness of the birth dose vaccination should target babies delivered during weekends and outside the hospital facility. Inclusion of immunization into the health education curriculum of schools would improve knowledge of its importance.

## Additional Files

The Additional Files for this article can be found as follows:

10.5334/aogh.725.s1 Table 3A. Factors associated with presentation within day 7 for birth dose vaccination.

10.5334/aogh.725.s1 Table 3B. Factors associated with presentation within day 14 for birth dose vaccination.
