# Supplementary material for: Determinants of Timely Presentation for Birth Dose Vaccination at an Immunization Centre in North-central Nigeria
Source: Ann Glob Health. 2019 Mar 1;85(1):20. doi: 10.5334/aogh.725 (PMC7052312; doi:10.5334/aogh.725)
Supplement: Table 3A. — Factors associated with presentation within day 7 for birth dose vaccination. [file agh-85-1-725-s1.pdf]

Supplementary Tables

Table 3A: Factors associated with presentation within day 7 for birth dose vaccination

Parameter	Time to presentation ≤ day 7 N = 423 n (%)	> day 7 N = 59 n (%)	Crude OR (95% C.I.)	p*	Adjusted OR (95% C.I.)	p**
**Gender**						
Male	218 (89.0)	27 (11.0)	1.27 (0.74–2.20)	0.386		
Female	203 (86.4)	32 (13.6)				
**Religion**						
Islam	301 (86.7)	45 (13.3)	0.78 (0.41–1.47)	0.444		
Christianity	120 (89.6)	14 (10.4)				
**Mother's age (years)**						
Mean (SD)	28.48 (4.72)	26.81 (4.35)	-	0.007 $^{\delta}$	1.08 (1.01–1.16)	0.021
**Mother's level of education**						
Postsecondary	288 (93.5)	20 (6.5)	4.22 (2.37–7.52)	< 0.001	2.75 (1.44–5.26)	0.002
≤Secondary	133 (77.3)	39 (22.3)			1 (reference)	
**Social class of child**						
Upper (I, II)	248 (91.5)	23 (8.5)	2.24 (1.28–3.92)	0.004	1.23 (0.64–2.35	0.531
Lower (III, IV)	173 (82.8)	36 (17.2)				
**Birth order of infant**						
First	169 (86.2)	27 (13.8)	0.79 (0.46–1.34)	0.411		
≥ Second	252 (88.7)	32 (11.3)				
**ANC**						
Yes	405 (90.2)	44 (9.8)	8.63 (4.00–18.64)	< 0.001	3.03 (1.06–8.65)	0.039
No	16 (51.6)	15 (48.4)			1 (reference)	
**Hospital delivery**						
Yes	394 (90.6)	41 (9.4)	6.4 (3.25–12.61)	< 0.001	3.17 (1.12–8.11)	0.016
No	27 (60.0)	18 (40.0)			1 (reference)	
**Correctly states NPI schedule**						
Yes	217 (88.9)	27 (11.1)	1.26 (0.73–2.18)	0.405		
No	204 (86.4)	32 (13.6)				

δ = p-value derived from independent sample t-test; * = chi-square derived p-value: ** = p-value derived from logistic regression analysis; OR (95%C.I.) = odds ratio 95% confidence interval

Table 3B: Factors associated with presentation within day 14 for birth dose vaccination

Parameter	Time to presentation ≤ day 14 N = 454 n (%)	Time to presentation > day 14 N = 26 n (%)	Crude OR (95% C.I.)	p*	Adjusted OR (95% C.I.)	p**
**Gender**						
Male	232 (94.7)	13 (5.3)	1.05 (0.47–2.30)	0.913		
Female	222 (94.5)	13 (5.5)				
**Religion**						
Islam	328 (94.8)	18 (5.2)	1.16 (0.49–2.73)	0.738		
Christianity	126 (94.0)	8 (6.0)				
**Mother's age (years)**						
Mean (SD)	28.48 (4.74)	26.92 (3.97)	-	0.108 $^{\delta}$		
**Mother's level of education**						
Post-secondary	300 (97.4)	8 (2.6)	4.38 (1.86–10.31)	< 0.001	3.10 (1.25–7.71)	0.015
≤Secondary	154 (89.5)	18 (10.5)			1 (reference)	
**Social class of child**						
Upper (I, II)	259 (95.6)	12 (4.4)	1.55 (0.70–3.43)	0.276		
Lower (III, IV)	195 (93.3)	14 (6.7)				
**Birth order of infant**						
First	181 (92.3)	15 (7.7)	0.49 (0.22–1.08)	0.072		
≥ Second	273 (96.1)	11 (3.9)				
**ANC**						
Yes	432 (96.2)	17 (3.8)	10.4 (4.17–25.94)	< 0.001#	5.66 (1.30–24.55)	0.021
No	22 (71.0)	9 (29.0)			1 (reference)	
**Hospital delivery**						
Yes	418 (96.1)	17 (3.9)	6.15 (2.56–14.77)	< 0.001#	2.15 (0.51–9.00)	0.297
No	36 (80.0)	9 (20.0)			1 (reference)	
**Correctly states NPI schedule**						
Yes	236 (96.7)	8 (3.3)	2.44 (1.04–5.72)	0.035	3.59 (1.38–9.31)	0.009
No	218 (92.4)	18 (7.6)			1 (reference)	

δ=p-value derived from independent sample t-test; *=chi-square derived p-value: **=p-value derived from logistic regression analysis; OR (95%C.I.)= Odds Ratio 95% confidence interval; #=Fisher exact test done.
